# Sensor Applications of Soft Magnetic Materials Based on Magneto-Impedance, Magneto-Elastic Resonance and Magneto-Electricity

**DOI:** 10.3390/s140507602

**Published:** 2014-04-25

**Authors:** Alfredo García-Arribas, Jon Gutiérrez, Galina V. Kurlyandskaya, José M. Barandiarán, Andrey Svalov, Eduardo Fernández, Andoni Lasheras, David de Cos, Iñaki Bravo-Imaz

**Affiliations:** 1 Department of Electricity and Electronics, Basque Country University (UPV/EHU), P.O. Box 644, 48080 Bilbao, Spain; E-Mails: jon@we.lc.ehu.es (J.G.); galina@we.lc.ehu.es (G.V.K.); manub@we.lc.ehu.es (J.M.B.); andrey.svalov@ehu.es (A.S.); eduardo.fernandez@ehu.es (E.F.); andoni.lasheras@ehu.es (A.L.); 2 BCMaterials, Technological Park of Biscay, N° 500, 48160 Derio, Spain; 3 ESS Bilbao, Paseo de Landabarri, 48940 Leioa, Spain; E-Mail: dce@we.lc.ehu.es; 4 Intelligent Information Systems, IK4-Tekniker, 20600 Eibar, Spain; E-Mail: inaki.bravo@tekniker.es

**Keywords:** magneto-impedance, magneto-elasticity, magneto-electricity, magnetic sensors

## Abstract

The outstanding properties of selected soft magnetic materials make them successful candidates for building high performance sensors. In this paper we present our recent work regarding different sensing technologies based on the coupling of the magnetic properties of soft magnetic materials with their electric or elastic properties. In first place we report the influence on the magneto-impedance response of the thickness of Permalloy films in multilayer-sandwiched structures. An impedance change of 270% was found in the best conditions upon the application of magnetic field, with a low field sensitivity of 140%/Oe. Second, the magneto-elastic resonance of amorphous ribbons is used to demonstrate the possibility of sensitively measuring the viscosity of fluids, aimed to develop an on-line and real-time sensor capable of assessing the state of degradation of lubricant oils in machinery. A novel analysis method is shown to sensitively reveal the changes of the damping parameter of the magnetoelastic oscillations at the resonance as a function of the oil viscosity. Finally, the properties and performance of magneto-electric laminated composites of amorphous magnetic ribbons and piezoelectric polymer films are investigated, demonstrating magnetic field detection capabilities below 2.7 nT.

## 1. Introduction

Magnetic sensors were introduced long ago to the field of electrical engineering and biomedical research. There are many types of magnetic field sensors. Many topical reviews have summarized the most important parameters and properties of magnetic field sensors, like bandwidth, full scale range, linearity, hysteresis, temperature coefficient of sensitivity, bias stability, offset features, long term stability, noise, resistance to the environment factors, power consumption, size, cost, *etc.* A detailed classification of the magnetic sensors based both on the employed materials and effects is given in [1–3]. For magnetic sensors based on semiconductors, superconducting quantum interferometers (SQUIDs) and recording heads see also [4,5]. Interesting examples of more specific descriptions of selected types of magnetic sensors based on the Hall effect, fluxgate sensing, magnetoresistance, giant magnetoimpedance (MI), and spin-polarized can be found in [2,6–8].

The coupling between the magnetic properties of soft magnetic materials and their elastic or electrical response gives rise to different phenomena that can be used for sensing purposes. On the one hand, the magneto-impedance (MI) accounts for the skin-effect-induced change of impedance caused by an applied magnetic field. It allows developing sensible magnetic field sensors for applications such as geomagnetism and biosensing, among many others. On the other hand, magnetoelastic resonance (MR) takes place when a mechanical resonant response is excited through the magnetostrictive effect of soft magnetic materials. The effect can be used to sense any magnitude that influences the resonant behavior and, as the resonance can be excited remotely, this effect is very convenient for non-contact applications. Finally, by combining those magnetostrictive amorphous ferromagnetic ribbons with piezoelectric materials, we have fabricated magneto-electric (ME) laminated composites, that show an extremely high sensitivity for magnetic field detection.

In close collaboration with the BCMaterials Research Center, and other Technological Centers of the Basque Country, the Group of Magnetism and Magnetic Materials of the Universidad del País Vasco (UPV/EHU) maintains an intense activity devoted to the development of sensors based on the above-mentioned magnetic materials and effects. The more recent results are described, for each of the research lines, in the following sections.

## 2. Magneto-Impedance (MI) Sensors

The magneto-impedance (MI) is the change of the complex impedance (*Z*) that ferromagnetic conductors experience when an external magnetic field (*H*) is applied and a high frequency (ω) alternating current flows through them. Great efforts were made in the last years in the development of magnetic thin-film-based sensitive elements with controllable properties for MI applications, including low magnetic field detection and biosensing [9–11]. Some excellent reviews are available [12–14]. One of the most studied MI thin film materials is the Fe_19_Ni_81_ Permalloy (Py), a soft magnetic material with low coercivity, high magnetic permeability and saturation magnetization [15]. Many sensor applications (for example, flat inductors, magnetoelectronic devices and MI itself) require FeNi thin film elements with thickness of the order of microns to obtain the desired properties [16–18]. In particular, MI is enhanced in sandwiched structures with a central non-magnetic conductor [19]. The magnetic softness of thin Py films depends on their thickness due to the crucial role of a transition into a “transcritical” state [18,20]. It manifests by the appearance of a perpendicular magnetic anisotropy component, high coercivity, and striped magnetic domain structure [21]. Different research groups have proposed the nanostructuring of Py films in order to solve this problem [11,18]. The effectiveness of using magnetic [Py/X] *_n_* multilayers (X—is a separating layer made of Cu, Ti, Ag or Gd and *n* the number of layers in the multilayered structure) was carefully analyzed [11,18,22,23]. This strategy was also successfully applied for Fe_72.5_Cu_1.1_Nb_1.9_Mo_1.5_Si1_4.2_B_8.7_ alloys [24]. In contrast, the thickness of the magnetic layers in the [Py/X] *_n_* multilayers has not been systematically analysed yet, although some interesting structural features observed in Py nanostructures can be used for the improvement of their magnetic properties [25].

In this section, we describe the preparation and characterization of MI samples containing Py/Ti multilayer structures with different Py layer thicknesses. The samples have a sandwiched structure, using a layer of Cu as central conductor between two Py/Ti multilayer structures. Although the Py layers have different thickness, the same total thickness of the top and bottom structures of the MI sandwich is maintained. We aim to obtain very high MI sensitivity with respect to the applied magnetic field systematically, with the purpose of designing optimized nanostructured multilayers sensitive elements to detect small magnetic fields.

### 2.1. Magneto-Impedance Sample Preparation

The multilayer sandwiched (MS) structures, where the magnetic layers are composed of Py/Ti multilayers, and the central non-magnetic layer is made of copper, were deposited by Direct Current (DC) magnetron sputtering onto glass substrates. The role of the background and working Ar pressure was previously studied to obtain the optimum deposition conditions [21]. In the present studies, a background pressure of 3 × 10^−7^ mbar and working Ar pressure of 3.8 × 10^−3^ mbar were used. The [Py/Ti] *_n_* magnetic layers at the top and bottom of the sandwiched structures were composed of equal thickness Fe_80_Ni_20_ layers, separated by 6 nm Ti layers. The thickness of the Ti spacer was optimized in a previous study of FeNi/Ti/FeNi bilayers [23]. In all cases, the sum of the thickness for all the Py layers in the structure was equal to 300 nm. The number (*n*) of Py and Ti layers in the multilayer was adjusted accordingly.

**Figure 1.**
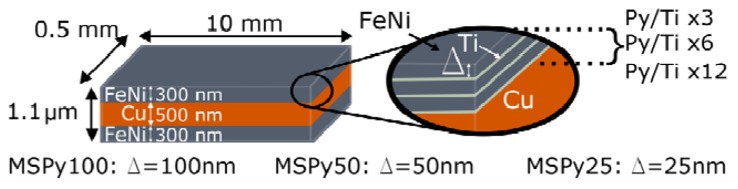
Schematic description of the Magneto-Impedance multilayer sandwiched (MS) structure used in this work. Each sample has different Py thickness (Δ) and different number of repetitions of the [Py/Ti] structure (3, 6 and 12 for samples MSPy100, MSPy50, and MSPy25, respectively), being the Cu central conductor of 500 nm in all cases.

A transverse magnetic anisotropy was induced during the deposition process by the application of an in-plane constant magnetic field of 250 Oe. The samples were shaped during deposition as 10 mm long and 0.5 mm wide rectangles using metallic masks. The long sides of the samples were oriented perpendicularly to the direction of the applied magnetic field. Therefore, the induced magnetic anisotropy axis was formed parallel to the short side of the stripe in all samples. Figure 1 and Table 1 give a description of the studied MS structures.

The in-plane hysteresis loops were measured using a vibrating sample magnetometer (VSM). The importance of the dispersion of the easy magnetization axes was widely discussed in the literature for the case of MI in amorphous ribbons [26]. In this work, fine features of the transverse magnetic anisotropy induced in Py/Ti multilayers were estimated following the procedure described in [27], *i.e.*, by measuring the sample's second harmonic magnetic response to the application of a small amplitude Alternating Current (AC) exciting field, with decreasing and increasing fields denoted as “Down” and “Up”. Even in the case of wide anisotropy distributions, the measured experimental distribution parameters give useful information about the magnetization processes in the multilayered structures.

The MI measurements were performed using Radio Frequency (RF) techniques, which are described in detail elsewhere [28]. The samples were glued into a 50 Ω microstrip line using silver paint. The total impedance (*Z*) and the real (*R*) component were obtained from the measured *S*_11_ parameters after calibration and mathematical subtraction of the test fixture contributions. The test fixture was placed inside the central region of a pair of Helmholtz coils and the full cycle of the impedance change was measured for the interval ±150 Oe of the external applied magnetic field. An exciting current in the frequency range of 300 kHz to 300 MHz was used. The frequency of 300 MHz is low enough to assure that resonance effects were not present and that the quasi-static processes dominated the MI behavior [29]. The MI ratios Δ*Z/Z*_sat_ and *ΔR/R*_sat_ were defined with respect to the magnetic field *H*_sat_ = 150 Oe, at which the samples were magnetically saturated. MI was defined as follows: *ΔZ*/*Z*_sat_ = 100 × [*Z*(*H*) − *Z*(*H*_sat_)]/*Z*(*H*_sat_) for the total impedance and *ΔR*/*R* = 100 × [*R*(*H*) − *R*(*H*_sat_)]/*R*(*H*_sat_) for the real component of the impedance. The MI sensitivities were defined as *s* [(*ΔZ*/*Z_sat_*)/*ΔH*] and *s* [(*ΔR*/*R*_sat_)/*ΔH*], with *ΔH* = 0.1 Oe. The direct current (DC) resistance *R*_DC_ was measured by the four contacts technique.

### 2.2. MI Results and Discussion

**Figure 2.**
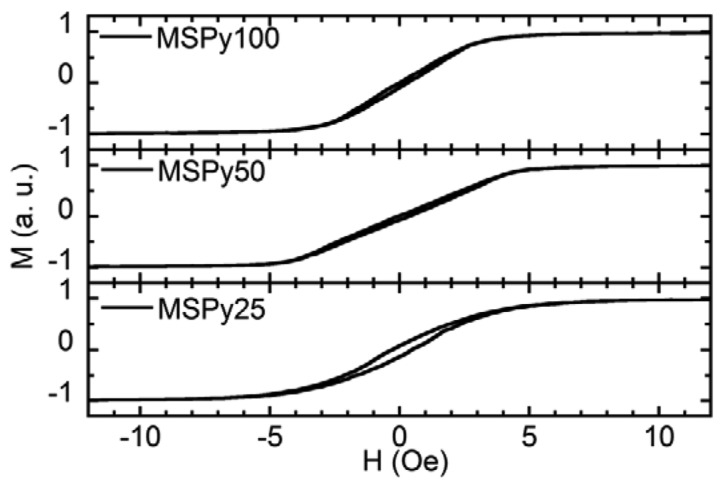
Vibrating sample magnetometer (VSM) hysteresis loops measurements, with the applied magnetic field perpendicular to the easy axis.

**Figure 3.**
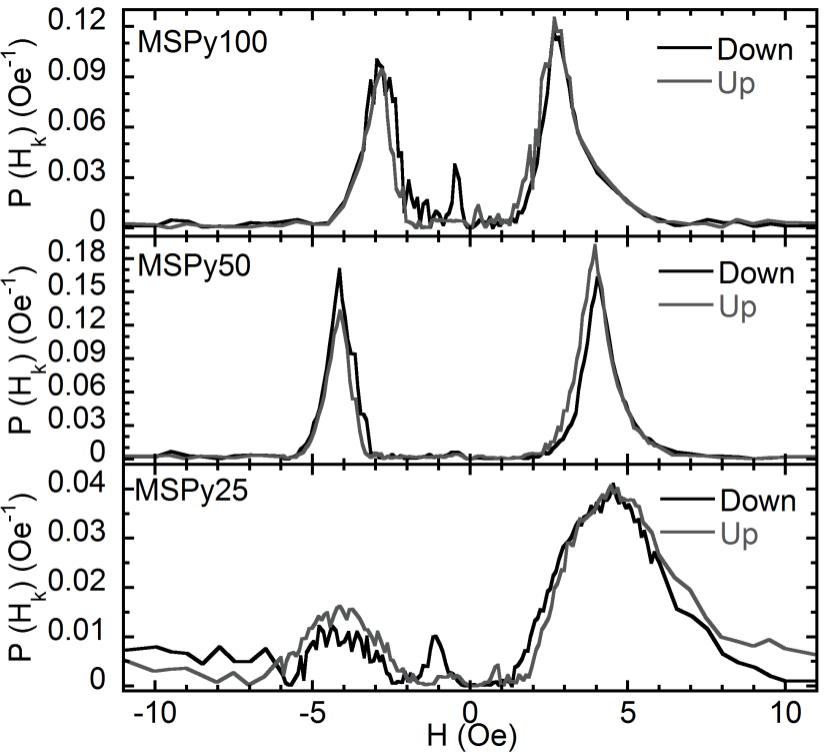
Distribution of perpendicular anisotropies from the second harmonic response.

Figure 2 shows the hysteresis loops of the MS structures obtained with a VSM. The magnetic field was applied in plane and along the short side of the rectangular sample, *i.e.*, along the easy magnetization axis. The shape of the hysteresis loops confirms the formation of an in-plane, transverse induced magnetic anisotropy. All the MS structures are very soft ferromagnets with well-defined magnetic anisotropy, close to uniaxial. Sample MSPy50 (Py layers 50 nm thick) has the smallest coercive field of 0.15 Oe. Samples MSPy100 and MSPy25, composed of 25 and 100 nm thick Py layers, have respectively coercive fields of 0.4 Oe and 0.2 Oe. The anisotropy fields (*H_k_*), listed in Table 1, have been obtained from the anisotropy distribution measurements corresponding to the maxima of the anisotropy distribution shown in Figure 3. These values are in close agreement with the anisotropy fields estimated from the *M*(*H*) hysteresis loops measured by VSM (Figure 2). Sample MSPy50 has a narrower anisotropy distribution than sample MSPy100, with larger intensity peaks, meaning that the sample has a better defined transverse anisotropy.

Several factors influence the magnetic behavior of the samples and must be considered. On the one hand, the magnetic properties of each individual layer must be taken into account. The type of domain walls depends on the thickness of the Py films as studied in [25,30]. A single layered 100 nm thick Py film displays two types of domain walls: 180 Néel walls with cross ties and stray-field free asymmetric mobile-vortex Bloch walls. In the 50 nm and 25 nm thick films the magnetic domains with in-plane orientation of the magnetization are separated by Néel walls with cross-ties. The different type of domain wall may surely influence the interaction between layers, as discussed in [31] for the case of bilayers. The advantages of weak interlayer interactions for maintaining a good magnetic softness are evident for certain thicknesses of the non-magnetic spacer.

On the other hand, the different contributions of the shape anisotropy to the effective anisotropy of the narrow [Py/Ti] *_n_*/Cu/[Py/Ti] *_n_* (*n* = 3, 6, 12) stripes can play a decisive role. The easy magnetization axis (EMA) induced during preparation is oriented along the short side of the stripe. The induced anisotropy is therefore competing with the shape anisotropy for which the minimum free energy corresponds to the position along the sample's length [32]. The results of the magnetic measurements confirmed that the mean anisotropy field is larger for the multilayers with thinner Py layers (and larger number of layers).

**Figure 4.**
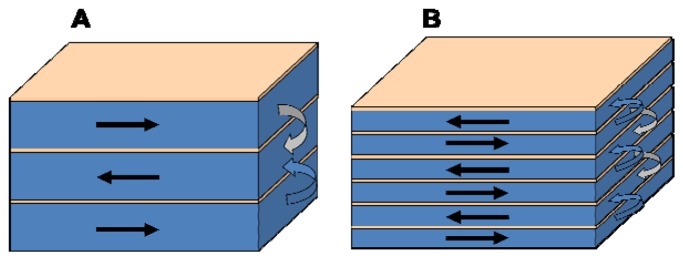
Schematic view of the closing magnetic flux at the lateral edges of the [Py(100 nm)/Ti] _3_ and [Py(50 nm)/Ti] _6_ multilayers.

Moreover, as schematized in Figure 4, each magnetic layer except the first and the last, have two nearest neighbors to close the magnetic flux and decrease the density of the magnetostatic energy at the surfaces perpendicular to the EMA. As the number of layers increases, this circumstance is more favorable, increasing the magnetic field that is necessary in order to rotate the magnetization towards the hard magnetization axis. At the same time, a large number of interfaces is always a source of magnetic inhomogeneities as can be seen from the analysis of the anisotropy distribution of sample MSPy25 in Figure 3.

**Figure 5.**
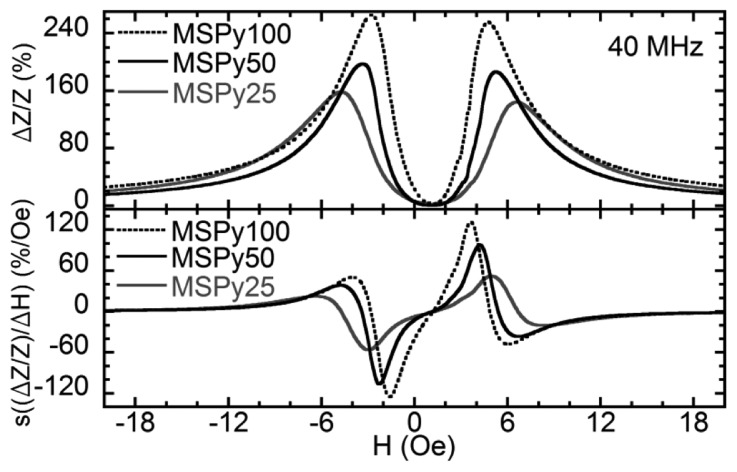
Relative magneto-impedance and sensitivity as a function of the applied magnetic field at 40 MHz.

Figure 5 displays the MI ratios and sensitivities obtained for the MS structures as a function of the external magnetic field at 40 MHz. The two-peaked shape of the MI curves is in accordance with the magnetic measurements. Samples MSPy50 and MSPy25 have similar anisotropy fields, so their MI ratios should be similar if we take into account only the MI dependence on the transverse magnetic permeability. Besides, sample MSPy25 has a smaller *R*_DC_ = 2.25 Ω compared to the 2.51 Ω of sample MSPy50. The MI ratio would be higher for sample MSPy25 with both samples having the same Δ*Z* value. Despite all the advantages that sample MSPy25 should have, at 40 MHz, sample MSPy50 shows a higher MI ratio compared with sample MSPy25. The MI sensitivity measured for the sample MSPy50 is also larger than in sample MSPy25. This is related to the higher peak widths of the anisotropy distribution of MSPy25 sample (Figure 3) that makes its MI peak less sharp and therefore decreases the sensitivity. Sample MSPy100 has the best MI response due to the combination of low anisotropy field and rather narrow anisotropy distribution. A lower anisotropy field implies a larger transverse magnetic permeability that increases the MI ratio and MI sensitivity. The maximum MI ratio in sample MSPy100 is indeed larger than in sample MSPy50.

**Figure 6.**
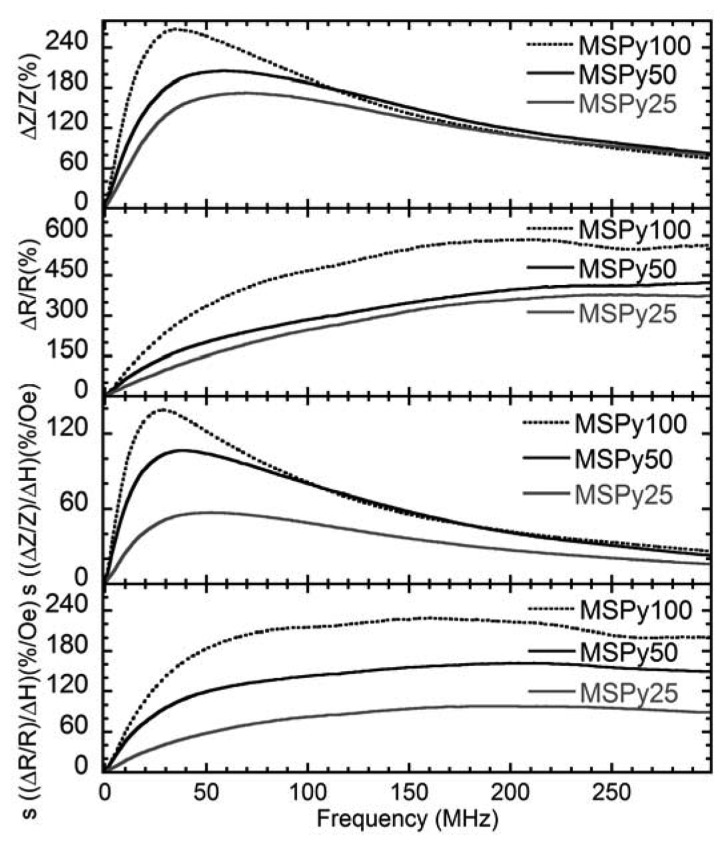
Frequency dependence of the maximum value of the Δ*Z*/*Z* ratio, maximum value of the Δ*R*/*R* ratio, maximum value of the sensitivity *s* [(Δ*Z*/*Z*)/Δ*H*] and maximum value of the sensitivity of the real part of the impedance *s* [(Δ*R*/*R*)/Δ*H*] for the three multilayer sandwiched structures.

The change in impedance Δ*Z* at 40 MHz is slightly larger in sample MSPy50 than in sample MSPy100. However, sample MSPy100 has a smaller DC resistance of 1.65 Ω which makes its impedance variation larger. The MI sensitivity is larger in sample MSPy100 than in MSPy50, as it can be expected since sample MSPy100 has a larger MI ratio and lower anisotropy field. The behavior of the maximum values of the MI ratio and the MI sensitivities in the whole range measured frequencies are shown in Figure 6. All the samples show quite similar behavior. The MI ratios at frequencies over 200 MHz are almost the same for all cases under consideration. At frequencies below 200 MHz, sample MSPy100 has the best MI response and MI sensitivity. The maximum sensitivity of 225%/Oe at 160 MHz was observed for the real part of the impedance.

It is interesting to compare the results obtained with previous data on MI of MS structures with thicker magnetic layers and the same thickness of the Cu lead. A maximum MI ratio of about 270% at 35 MHz and a maximum sensitivity of about 140%/Oe at 30 MHz are obtained for sample MSPy100. These values are larger than the ones obtained for the MS structure described in [28] where the thickness of each Py layer was 170 nm and the total thickness of each magnetic layer was 500 nm. The samples were deposited in the same conditions and with the same type of MS structure (length, width and central conductor thickness) as in the present work. Despite of the lower total thickness of the ferromagnetic parts of the multilayer, sample MSPy100 has higher MI ratio and MI sensitivity to the applied field.

Finally, one can expect further increase of the MI ratio and the MI sensitivity of the MI multilayers of MSPy100, MSPy50 and MSPy25 type with an increase of the total thickness of the ferromagnetic layers to 500 nm, *i.e.*, matching the thickness of the central Cu layer, as it seems to be the best geometrical configuration leading to the highest MI results [17,31].

Of course, from the production point of view, thicker Py layers forming a multilayered structure are always an advantage due to the less number of technological steps for the deposition. Smaller number of the interfaces is also favorable because it was shown that mixed interfaces could be a reason for the decrease of the magnetic softness and the increase of anisotropy distribution width [18,25]. At the same time there are some important topological implications, for the case of the multilayered structures only, which have not been discussed in the literature yet. One of these topological aspects is whether there is an even or odd number of the Py layers in the [Py/Ti] *_n_* or [Py/Cu] *_n_* multilayered structure. The even/odd configuration can dramatically change the conditions at which the magnetic flux is closed, being especially important for miniaturized MI sensitive elements obtained by lithography. Research in this direction is being planned.

### 2.3. MI Conclusions

Magneto-impedance sandwiched structures consisting of a Cu central layer between two Py/Ti multilayered films were prepared by DC sputtering. In each structure, a different thickness of the Py layers was used (25, 50 and 100 nm) while maintaining the same total thickness of 300 nm for the magnetic material, by using the corresponding number of Py/Ti repetitions. It was found that the sandwiched structure composed of 100 nm thick Py layers present a better performance than the one with 50 nm thick Py layers and much better than the one with 25 nm thick Py layers. The best results obtained for the [Py/Ti] _3_/Cu/[Ti/Py] _3_ sandwich structure were: MI ratio of 270% at 35 MHz and a sensitivity of 140%/Oe at 30 MHz. These values indicate the capabilities of optimized nanostructured multilayers to be competitive materials for small magnetic field sensitive elements well integrated into semiconductor electronics.

## 3. Magnetoelastic Resonance Sensor

The coupling between the magnetic and elastic properties of ferromagnetic materials gives rise to a number of physical effects that can be used for sensing purposes. The devices that make use of them are denominated magnetoelastic sensors. The magnetostrictive effect accounts for the change of dimensions produced in a magnetic material upon magnetization. The reverse effect, that is, the change in the magnetic state of a material caused by the application of a mechanical stress, is known as the magnetoelastic effect (also called inverse magnetostrictive or Villari effect). The magnetoelastic effect can be readily used to sense stress or any other related magnitude, and its fundamental advantage over other principles of sensing is it intrinsic non-contact nature, since magnetization changes can be detected inductively. Examples of the application of the magnetoelastic effect into successful commercial devices are described elsewhere [33].

Since in a magnetostrictive material, stress and magnetization are intimately coupled, the stress perturbation caused by a sound wave traveling in the material is accompanied by a magnetization one, giving rise to a magnetoelastic wave, whose mathematical formalism is treated exhaustively in [34]. Under adequate circumstances, the traveling magnetoelastic waves become stationary, giving rise to resonances. For example, when a magnetic sample of length *L* is magnetized by an alternating magnetic field, if the wavelength of the magnetoelastically induced sound wave matches the dimension of the sample, a resonance is built up, which is called magnetoelastic resonance. For a sample of density ρ, having a Young modulus *E*, oscillating with free ends, the resonant frequency is given by:

(1) $$f_{r}=\frac{1}{2L}\sqrt{\frac{E}{\rho }}$$

**Figure 7.**
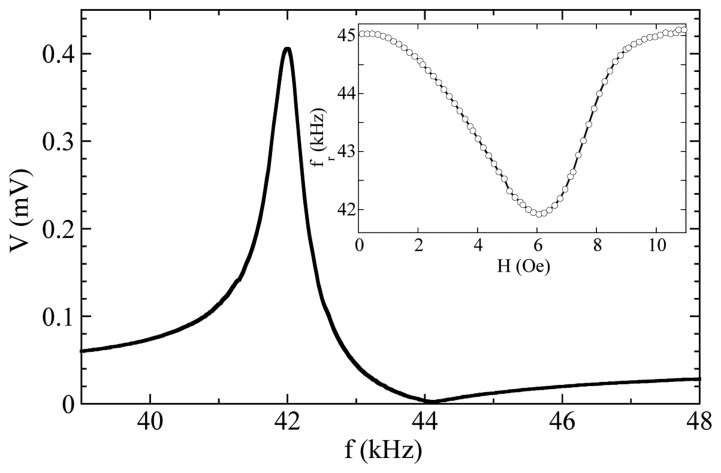
Magnetoelastic resonance of an amorphous ribbon. The inset shows the dependence of the resonant frequency on the applied magnetic field.

At resonance, great magnetization changes takes places and the permeability of the sample, that can be detected inductively using a coil wounded around the sample, increases greatly. Figure 7 displays a typical magnetoelastic resonance measured in a commercial amorphous ribbon 5 cm long. It is especially important to state that, due to the magnetoelastic coupling, the value of the Young's modulus *E* depends on the magnetic state of the sample (this is the so-called Δ*E* effect). Therefore, the frequency of resonance can be tuned by biasing the sample by a suitable magnetic field as exemplified in the inset of Figure 7.

With applications in mind, the magnetoelastic resonance can be used to remotely detect any parameter that affects the magnetostrictive element, such as temperature [35], or pressure [36]. The magnetoelastic material can be also covered by a chemically responsive layer to develop gas, humidity or pH sensors [37]. Probably the most successful application of the magnetoelastic resonance are the acousto-magnetic antishoplifting labels, whose principle of operation is described in [33]. In this work we describe the use of the magnetoelastic resonance to determine the viscosity of lubricant oils, through the damping caused by the oil in the oscillations of a magnetoelastic sample.

### 3.1. Experimental: Oil Viscosity Sensor

The roles of lubricant oils in machinery include not only friction reduction, but also refrigeration, segregation of contaminants and debris and protection against wear and corrosion. These tasks degrade the oil, so it must be replaced to prevent failures. For optimum performance, it is necessary to develop sensors that provide online and real-time measurements of the condition of the lubricant oil. The viscosity of the oil is severely affected by the degradation of the oil due to aging and/or contamination and, therefore, a sensor determining continuously the viscosity of the lubricant oil can help implementing a predictive maintenance strategy in machinery.

**Figure 8.**
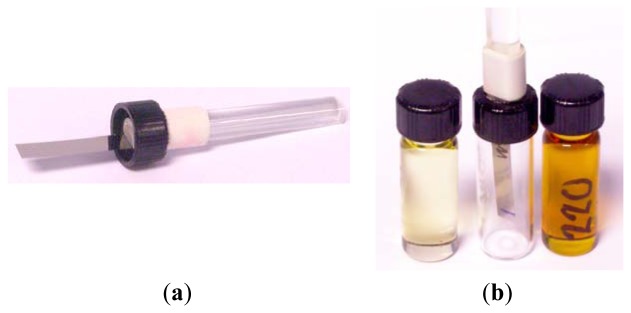
(**a**) The magnetoelastic sample is glued to a glass rod, fixed to the cap of the vial; (**b**) When the cap is fitted to the vial, the sample is immersed in the oil.

The amplitude and frequency of the magnetoelastic response of a material immersed in a viscous medium depends on the viscosity of such medium. Our prototype uses a commercial amorphous ribbon (VITROVAC 4040 kindly provided by Vacuumschmelze GmbH, Hanau, Germany), 33 mm long, 6 mm wide and 23 μm thick. One extreme of the sample is glued to a glass rod and inserted in the oil under test, which fills a 4 mL vial (see Figure 8). For detecting the resonance, a two-coil system with separated excitation and detection coils is used. The detection coil is complemented with a compensation coil connected in opposition. The bias field to set the optimum magnetic state of the sample is provided by a pair of Helmholtz coils. The resonance measurements were performed using a spectrum analyzer performing a continuous frequency sweep.

Oils from two different families covering a wide range of viscosities were used for the experiments. Three samples of oil used in hydraulic systems had viscosities of 32.4, 67.1 and 108.2 cSt respectively. Two additional samples of high duty oils used in gearboxes had viscosities 218.2 and 325.9 cSt respectively. The viscosity of all the samples was determined using the ASTM D-445 procedure (American Society for Testing and Materials, ASTM International, West Conshohocken, PA, USA), within an accuracy of 0.1 cSt [38].

### 3.2. Data Analysis Procedure

To analyze the data we have developed a phenomenological approach to describe the frequency response of a magnetoelastic material. The magnetoelastic resonance is characterized by a sharp maximum at the resonance followed by a null minimum at the antiresonance (see Figure 7). Using the formalism of linear systems, an analytical expression for the transference function can be readily written: the resonance imposes a couple of complex conjugated poles in the denominator, and the antiresonance is described with a couple of complex conjugated zeros in the numerator. The transference function is then:

(2) $$G(s)=\frac{\omega_{r}^{2}}{\omega_{a}^{2}}\cdot \frac{s^{2}+2\delta_{a}\omega_{s}s+\omega_{a}^{2}}{s^{2}+2\delta_{r}\omega_{r}s+\omega_{r}^{2}}$$

where ω*_r_* is the value of the resonance frequency, ω*_a_* the antiresonance, and δ*_a_* and δ*_r_* are damping parameters. In this formalism *s* = *j*ω, where ω is a real frequency and $j=\sqrt{-1}$.

Experimentally, the measured resonance curves can be fitted to:

(3) $$V(\omega )=A\left|\frac{\omega^{2}-2j\delta_{a}\omega_{a}\omega -\omega_{a}^{2}}{\omega^{2}-2j\delta_{r}\omega_{r}\omega -\omega_{r}^{2}}\right|+a\omega +b$$

**Figure 9.**
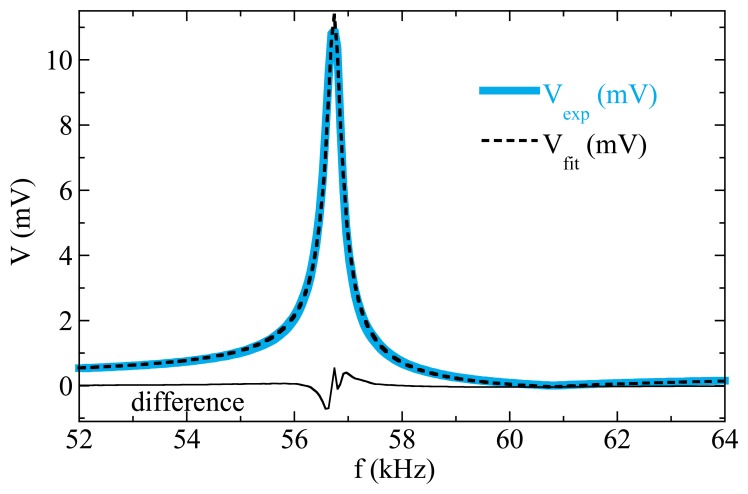
Fitting of a measured resonance curve to Equation (3). Best fit parameters are: *f_r_* = 56.73 kHz; δ*_r_* = 0.0022; *f_a_* = 60.82 kHz; δ*_a_* = 0.0121; *A* = 38.07 mV; 2π*a* = 8.57 × 10^−6^ mV/Hz; *b* = −55.97 mV.

The only parameters to be fitted are the resonance ω*_r_* = 2π*f_r_* and the antiresonance ω*_a_* = 2π*f_a_* frequencies, and the damping constants δ*_r_* and δ*_a_*, together with the parameters *A*, *a*, and *b* that account for the amplitude and a linear background respectively. Figure 9 shows the excellent agreement found by a least squares fit for the resonance curve measured in a 37 mm long, 6 mm wide VITROVAC ribbon.

### 3.3. Results and Discussion

**Figure 10.**
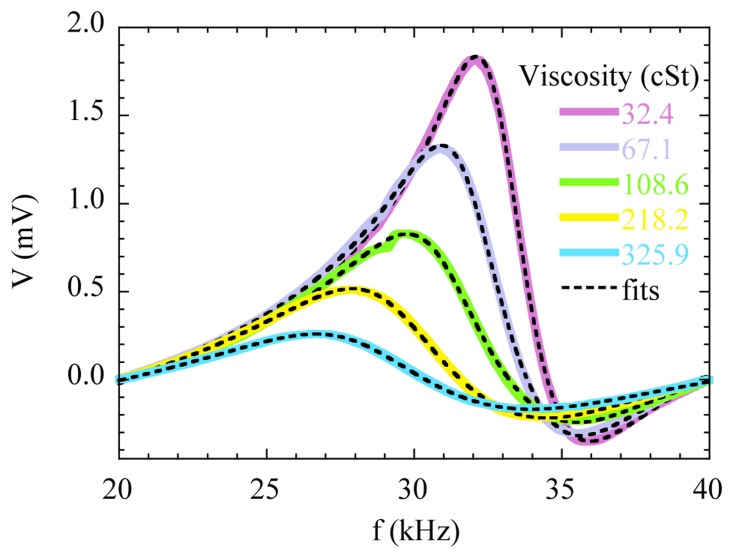
Magnetoelastic resonance curves measured for oils with different viscosities. The dashed lines correspond to the fitting of the experimental curves to Equation (2).

Figure 10 shows the resonance curves obtained with the magnetoelastic material immersed in oils with different viscosities. For better comparison, the linear background has been subtracted from the measured curves. All the curves were obtained at room temperature with an applied bias magnetic field of 6.7 Oe, which was checked to provide the maximum amplitude of the resonance in air. The AC excitation amplitude was set to about 0.1 Oe, which is relatively large to obtain a good signal to noise response, but still low to avoid non-linear effects [39].

As expected, the amplitude of the resonance curves systematically decreases as the viscosity of the oil increases, whereas the width of the resonance increases with the viscosity, due to the increasing damping. The resonance frequency and the amplitude can be extracted directly from the measured curves to obtain its dependence on viscosity [40]. Additionally, Figure 10 shows the fits of the data to Equation (1). The fitting results allow us to accurately determine these parameters, but also the damping parameter δ*_r_* intimately related to the viscosity.

**Figure 11.**
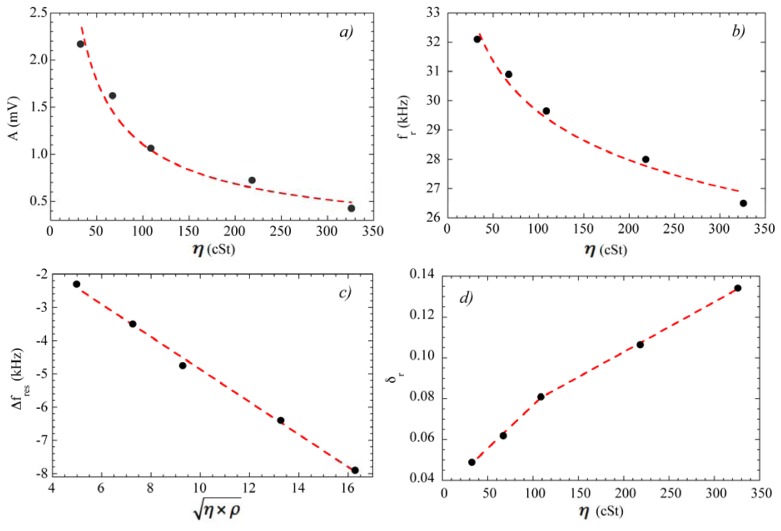
Dependence of best-fit parameters on oil viscosity η. (**a**) Amplitude of the resonance; (**b**) Resonance frequency; (**c**) The resonance frequency shift (from the resonance frequency in air) scales with the square root of the product of the viscosity times the density of the oil; (**d**) Damping parameter.

Figure 11 represents the results obtained from the fittings. Figure 11c shows that the frequency shift with respect to the resonance in air follows a linear relation with the square root of the product of the viscosity and the density of the oils, as predicted by the theory for low viscosity regimens [41]. The dependence of the damping parameter δ*_r_* on the viscosity reveals two distinct slopes that correlate with the different nature of the oils under test. This is an indication of the capabilities of the method for detecting fine features in the viscosity of the oils.

### 3.4. Conclusions and Outlook

The magnetoelastic resonance is revealed as a useful technique to determine the viscosity of lubricant oils in a wide range of viscosities, from 32 to 326 cSt. The fitting of the resonance curve to a phenomenological equation allows determining not only the dependence of the resonance frequency and the amplitude of the resonance on the viscosity, but also the damping parameter. Any of these parameters can be used for designing a sensor for on-line and real-time assessing the degradation of lubricant oils based on viscosity measurements. Further development will attempt to determine the viscosity from the time-dependent attenuation of the oscillations of the magnetoelastic samples after a pulsed excitation at the resonant frequency.

## 4. Magnetoelectric Sensor

The combination of magnetostrictive amorphous ferromagnetic ribbons with piezoelectric materials, allows obtaining magnetoelectric laminated composites, that show an extremely high sensitivity for magnetic field detection. Combinations of VITROVAC type magnetic alloys epoxyed to Polyvinylidene Fluoride (PVDF) piezoelectric polymer give as result magnetoelectric coefficients above 80 V/cm Oe. Also, high temperature new piezopolymers as polyimides are can be used for the magnetoelectric detection at temperatures as high as 100 °C.

### 4.1. Magnetoelectric Experimental Details

**Figure 12.**
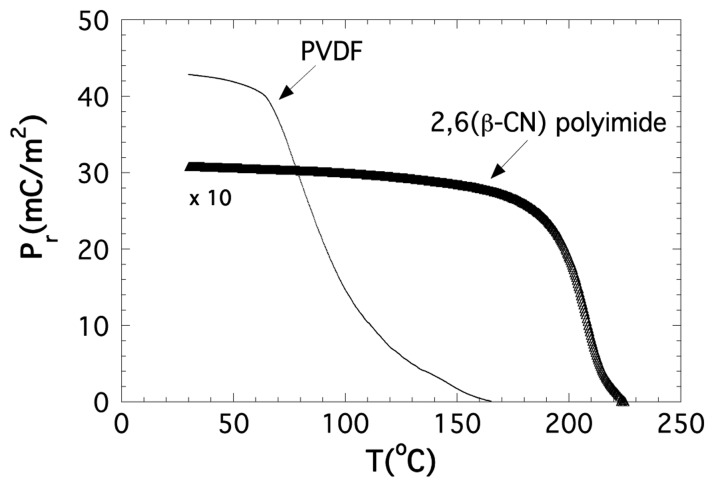
Measured remnant polarization as a function of temperature for commercial PVDF piezoelectric polymer and the new piezolelectric 2,6(β-CN)APB/ODPA (poly-2,6) polyimide.

We construct magnetoelectric three-layer sandwich-like with longitudinal magnetostrictive operation and transverse piezoelectric response (L-T type) laminated composites by gluing two equal magnetostrictive ribbons to opposite sides of polymer piezoelectric films with an adhesive epoxy resin (Figure 12, upper left). Magnetostrictive ribbons belonging to the family of Fe-Co-Ni-Si-B, Fe-rich metallic glasses have a measured magnetostriction that ranges between λ*_s_* ≈ 8–30 ppm and maximum value for the piezomagnetic coefficient *d_33_* = *d*λ*/dH* of about 0.6 − 1.5 × 10^−6^/Oe. This last parameter will modulate the magnetoelectric response of the composite as a function of the applied bias magnetic field.

Concerning the piezoelectric material we firstly used the well-known polymer PVDF, the well-known piezoelectric polymer [42], with glass transition and melting temperatures about −35 °C and 171 °C, respectively, but a Curie temperature of ≈100 °C. This makes its piezoelectric response to decay quickly above 70 °C (Figure 12). To develop a ME device being able to operate at higher temperatures, we have also tested new amorphous piezoelectric polymers of the family of the polyimides. Detailed information about synthesis, thermal characterization and electric polarization processes can be found in [43]. We only will remark briefly that its main parameters are a glass transition temperature of *T*_g_ ≈ 200 °C [44] and a degradation temperature of *T*_d_ ≈ 510 °C, temperatures that make these polyimides suitable for our purposes.

Taking advantage of the magnetoelastic resonance effect that enhances the magnetostrictive response, all measurements have been taken at resonance. For that, we first determine the static magnetic field *H*_DC_ necessary to induce the maximum amplitude of that resonance, using the measurement set-up previously described. Then, we measure the induced magnetoelectric voltage *V*_ME_ in the sandwich laminate (through two small silver ink contacts located at both opposite magnetostrictive ribbons) by the following procedure: under a *H*_AC_ magnetic excitation applied along the length axis, the magnetostrictive ribbons will elongate and shrink along the same direction. This will make the piezoelectric polymer film to undergo an AC longitudinal strain, inducing a dielectric polarization change in its transverse direction (L-T operation).

Thus, we can determine simultaneously (a) the magnetoelectric response dependence as the bias field *H*_DC_ changes; and (b) at the *H*_DC_ value for the maximum magnetoelastic resonance amplitude, the magnetoelectric voltage dependence *versus* the applied *H*_AC_ magnetic excitation, that is the so called *sensitivity* of the magnetoelectric composite.

From the measured induced magnetoelectric voltage, the magnetoelectric coefficient α_ME_ can be directly derived as [45]:

(4) $$\alpha_{ME}=\frac{dE_{1}}{dH_{3}}=\frac{1}{t}\left(\frac{\delta V_{ME}}{\delta H_{ac}}\right)$$

**Figure 13.**
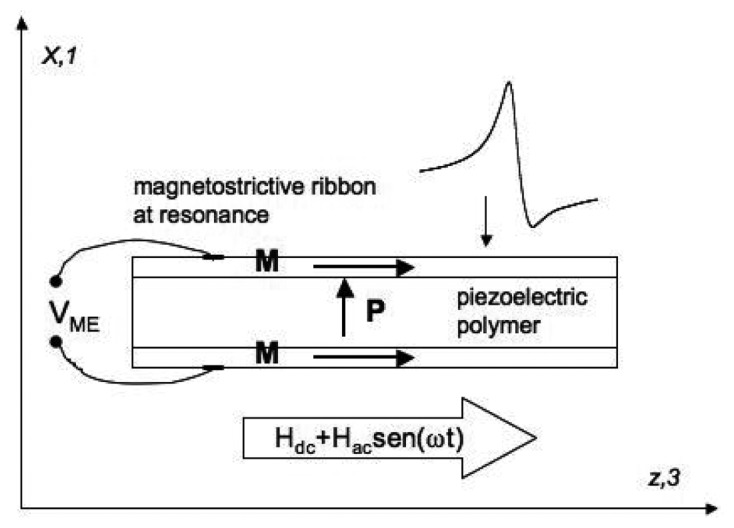
Geometry of the three-layer L-T sandwich configuration: magnetostrictive ribbons are longitudinally magnetized while the piezoelectric polymers were transversely poled.

In this equation, *t* is the thickness of the piezoelectric layer. For L-T operation (Figure 13) this coefficient can also be written as α_ME_ = α_31_.

### 4.2. Applications Based on the Magnetoelectric Effect

Applications based on the ME effect are as widespread as magnetic field sensors [46,47], current sensors [48], transformers [49], microwave devices [50] and FMR resonators and filters [51,52]. The highest ME response has been reported for laminated magnetostrictive/piezoelectric polymer composites. Fang *et al.* [53] reached a magnetoelectric voltage coefficient of 21.46 V cm^−1^ Oe^−1^ for a METGLAS 2605 SA1/PVDF (Metglas, Conway, SC, USA) laminate achieved at non-resonance frequencies and is, so far, the highest response obtained at sub-resonance frequencies. At the longitudinal Magnetoelastic Resonance (MER) of the magnetostrictive constituent, energy transference from magnetic to elastic, and *vice versa*, is maximum. This energy conversion at the resonance turns out to be very sharp for ME laminates, while frequency bandwidth for applications based in this MER enhancement effect remains limited. Jin *et al.* [54] reached a magnetoelectric voltage coefficient of 383 V cm^−1^ Oe^−1^ on cross-linked P(VDF-TrFE)/METGLAS 2605 SA1, the highest reported to date.

**Figure 14.**
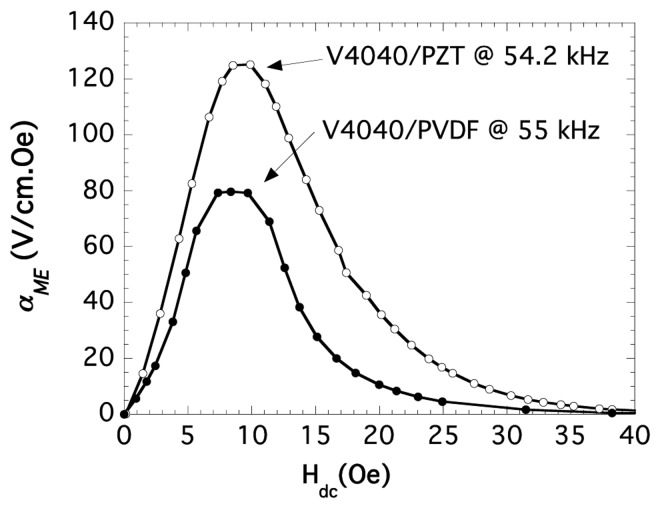
Measured α_ME_ magnetoelectric coupling coefficient for our L-T type laminated composite with VITROVAC 4040^®^ as magnetostrictive constituent and ceramic PZT or PVDF polymer as piezoelectric constituent. Sizes of the devices are almost the same, as hinted by their close resonant frequencies.

Our first measurements have been performed with laminated composites of the type VITROVAC 4040^®^/PZT or PVDF/VITROVAC 4040^®^. As can be seen in Figure 14 both type of laminates show a good magnetoelectric response (of 125 V cm^−1^ Oe^−1^ and 80 V cm^−1^ Oe^−1^ for Lead zirconium titanate (PZT) and PVDF containing laminates, respectively) at low applied magnetic fields (about 10 Oe, due to the anisotropy field of the VITROVAC ribbon).

We have also measured the sensitivity of the VITROVAC 4040^®^/PVDF/VITROVAC 4040^®^ device as a function of temperature, getting a decrease from 357 mV/Oe (sensitivity below 0.3 nT) at room temperature to 142 mV/Oe at 80 °C (sensitivity below 0.8 nT).

In order to avoid this observed sensitivity decrease when increasing temperature, we have fabricated the same L-T structured magnetoelectric laminates with the same magnetostrictive constituents but using a 40/60 copolyimide as high temperature piezoelectric constituent [55].

**Figure 15.**
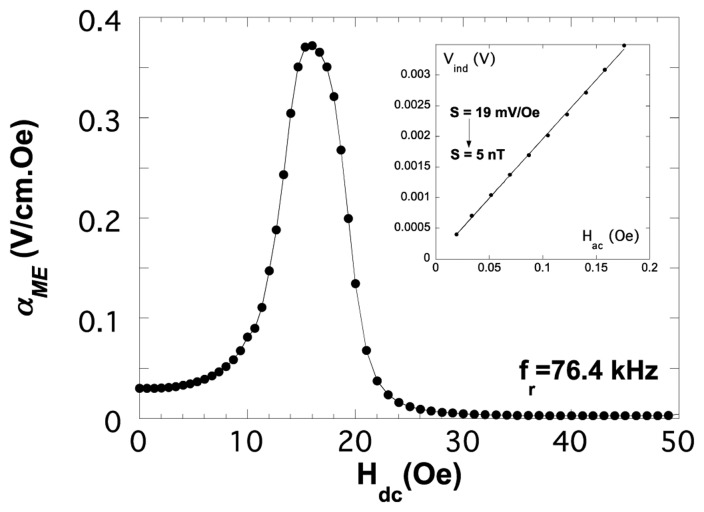
Room temperature measured α_ME_ magnetoelectric coupling coefficient for our L-T type laminated composite with METGLAS 2826 MB as magnetostrictive constituent and a 40/60 copolyimide as piezoelectric constituent. Reprinted with permission from [56]. Copyright 2013 IEEE *Xplore*.

As it can be seen in Figure 15 (Reprinted with permission from [56]. Copyright 2013 IEEE *Xplore*), even if the magnetoelectric response turns out to be much more modest than when using PVDF piezopolymer, the great advantage is that sensitivity keeps constant up to high temperatures >80 °C, with values about 37 mV/Oe (sensitivity below 2.7 nT).

Efforts to get wider bandwidths for MER and ME applications have been mainly based on magnetic field tuning procedures either in bimorph or tri-layered structures, but the maximum achieved frequency of operation has been some tenths of kHz [57]. Another way to get high frequencies of operation can be based on the relationship between length and resonant frequency value (*f_r_* ∝ 1/*L*) of magnetostrictive ribbons at the magnetoelastic resonance. So, our efforts are now focused on fabricating short magnetoelectric L-T type laminates showing good magnetoelectric response at high frequencies. Nevertheless, the higher the resonant frequency the lowest the amplitude of the resonance and as a first consequence, the magnetoelectric response will be also decreased. It is clear that a compromise between length of the device and so working frequency, and induced magnetoelectric signal, must be achieved.

**Figure 16.**
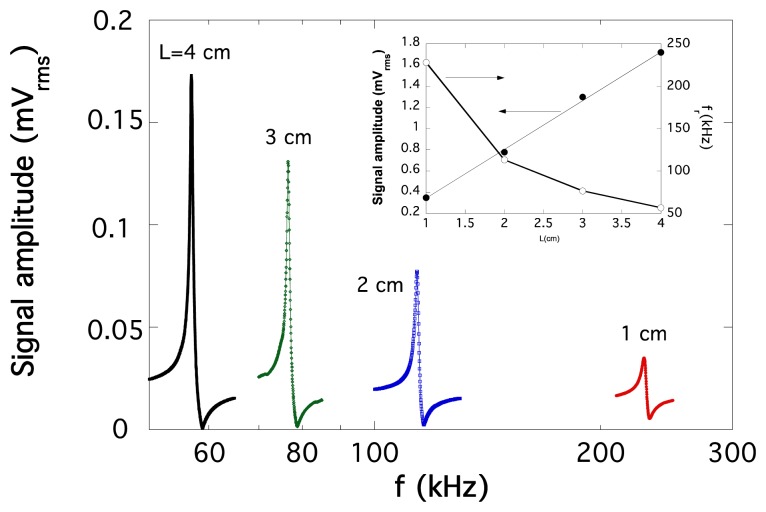
Magnetoelastic resonance curves measured for different ribbon pieces with length ranging from 4 cm to 1 cm. The inset shows also the measured resonance frequency and the induced signal at the resonance, as a function of the length of the resonant magnetostrictive ribbon.

Thus, we have developed a device 1 cm long for which the resonant (working) frequency rises to 230 kHz (See Figure 16), and the measured magnetoelectric coupling coefficient is about 15 V cm^−1^ Oe^−1^ when PVDF is used as piezoelectric constituent. Following this line of reasoning, we expect to construct a 0.5 cm long device that will work at a resonant frequency about 500 kHz. This fact, combined with the use of a high temperature piezopolymer as the polyimides previously described, can lead to a very useful class of magnetoelectric laminates working simultaneously at high temperature and within the radiofrequency range, both characteristics of great interest for low distance near field communications in aggressive environments (*i.e.*, the desert, a tunnel or fighting a fire).

### 4.3. Conclusions

Combining the excellent magnetoelastic response of magnetostrictive amorphous ferromagnetic ribbons with piezoelectric polymers, we have fabricated short length magnetoelectric laminated composites that show an extremely high sensitivity for magnetic field detection. We have already fabricated devices that work within the radiofrequency range and we expect to use them with high temperature new piezopolymers (polyimides) in order to achieve magnetoelectric detection at temperatures as high as 100 °C.

## 5. Conclusions/Outlook

Our recent research results on sensor technologies based on soft magnetic materials have been reviewed. First, the effect of the thickness of Py layers in multilayer sandwiched magneto-impedance structures has been studied. Second, the magnetoelastic resonance has been shown to produce useful results to determine the viscosity of lubricant oils, based on a new technique for analyzing the resonance curves. Finally, new magnetoelectric composites with excellent performance for low magnetic field detection have been described. The results presented in each technology constitute single steps in a long range research strategy of the Group of Magnetism and Magnetic Materials of the University of the Basque Country in Spain, aimed to develop high performance sensing devices based on soft magnetic materials.

## Figures and Tables

**Table 1.** Description of the samples and summary of selected properties.

Sample structure	[Py/Ti] _3_/Cu/[Ti/Py] _3_	[Py/Ti] _6_/Cu/[Ti/ Py] _6_	[Py/Ti] _12_/Cu/[Ti/ Py] _12_
Sample identifier	MSPy100	MSPy50	MSPy25
FeNi layer thickness	100 nm	50 nm	25 nm
*R*_DC_ (Ω)	1.65	2.51	2.25
*H_c_* (Oe)	0.15	0.20	0.40
*H_k_* (Oe)	2.8	4.0	4.5
(Δ*Z*/*Z*)_max_ (%)	270	210	170
(Δ*R*/*R*)_max_ (%)	580	420	380
*s* [(Δ*Z*/*Z*)/Δ*H*] _max_ (%/Oe)	140	110	60
*s* [(Δ*R*/*R*)/Δ*H*] _max_ (%/Oe)	230	160	100

*R*_DC_—DC resistance of the sample; *H_c_*—coercive field; *H_k_*—anisotropy field; (Δ*Z*/*Z*)_max_; (Δ*R*/*R*)_max_—maximum values of the MI ratio for the absolute value of the impedance and for the real part respectively. *s* [(Δ*Z*/*Z*)/Δ*H*] _max_, *s* [(Δ*R*/*R*)/Δ*H*] _max_—maximum sensitivities of both ratios.
